# IGF1 Binding to Integrin αvβ3 Induces Direct Gα13 Binding to IGF1R Kinase

**DOI:** 10.3390/ijms27094042

**Published:** 2026-04-30

**Authors:** Yoko K. Takada, Chun-Yi Wu, Yoshikazu Takada

**Affiliations:** 1 Department of Dermatology, University of California School of Medicine, Research III Suite 3300, 4645 Second Ave., Sacramento, CA 95817, USA; 2 Department of Neurology, University of California Davis School of Medicine, Sacramento, CA 95817, USA; chywu@health.ucdavis.edu; 3 Department of Biochemistry and Molecular Medicine, University of California School of Medicine, Research III Suite 3300, 4645 Second Ave., Sacramento, CA 95817, USA

**Keywords:** integrin, Gα13, IGF1, IGF1R, cell survival, outside-in signaling, Gα13 Q226L

## Abstract

IGF1 plays a critical role in cell proliferation and survival. Previous studies show that IGF1 binds to integrin αvβ3 and induces αvβ3-IGF1-IGF1R ternary complex formation. However, how IGF1 binding to αvβ3 leads to IGF1R activation is unclear. Previous studies showed that Gα13, a guanine nucleotide-binding protein of the G12 class of Gα proteins, binds to the integrin β3 tail through the EEE motif upon fibrinogen binding to integrin αIIbβ3 and induces RhoA activation. We discovered that the EEE/AAA mutation of the β3 tail inhibited IGF1-induced cell survival, suggesting that Gα13 binding to the β3 tail is required for IGF1 signaling. Since RhoA activation may not be directly involved in IGF1R activation, we studied if Gα13 binds to molecules other than RhoA. Since Gα13 binds to several cytoplasmic tyrosine kinases, we studied if Gα13 binds to the IGF1R kinase by a docking simulation. The simulation predicted that Gα13 binds to the IGF1R kinase through a new binding site. Mutating the predicted Gα13 binding site in the IGF1R kinase (residues 1020-1022) or the predicted IGF1R kinase binding site in Gα13 (residues 260-279) inhibited Gα13 binding to the IGF1R kinase, which is consistent with the docking model. Notably, the Gα13(260-279A) mutant inhibited IGF1-induced cell survival. We propose that IGF1 binding to αvβ3 induces Gα13 binding to the β3 tail and subsequent Gα13 binding to the IGF1R kinase, leading to IGF1R activation. Interestingly, Gα13(260-279A) mutation inhibited cell survival due to a constitutively active Gα13(Q226L) mutant. We propose that Gα13(Q226L) induces its effect by binding to the IGF1R kinase. We propose that the Gα13 binding site of the IGF1R kinase or the IGF1R binding site in Gα13 may be a novel therapeutic target.

## 1. Introduction

Insulin-like growth factor receptor type 1 (IGF1R) is a key member in the IGF axis and has long been recognized for its oncogenic role in multiple cancer lineages [1]. Many cancer cells secrete abnormally high levels of IGF1 and IGF2. Once released by cancer cells, both growth factors bind and activate IGF1R on the surface. It has been proposed that IGF1 binding induces phosphorylation of specific tyrosine residues of IGF1R. These phosphotyrosines then bind to adapter molecules such as Shc and insulin receptor substrate (IRS)-1. Phosphorylation of these proteins leads to activation of the phosphoinositide 3-kinase (PI-3K) and mitogen-activated protein (MAP) kinase signaling pathways [2]. This autocrine receptor activation causes the release of intracellular signals that are strongly anti-apoptotic, notably through their ability to activate the PI-3K/AKT pathway. Therefore IGF1 provides resistance to chemotherapy and radiation therapy. Thus, IGF1R is a major therapeutic target for cancer [3].

Integrins are a family of cell adhesion receptors that are transmembrane α-β heterodimers, and at least 18 α and 8 β subunits are known [4]. Integrins recognize extracellular matrix ligands (e.g., fibronectin and fibrinogen), cell surface ligands (e.g., VCAM-1 and ICAM-1) and soluble ligands (e.g., insulin-like growth factor-1, IGF1) [5]. Integrins are involved in signal transduction upon ligand binding (outside-in signaling) and are regulated by signals from inside the cells (inside-out signaling) [4]. Integrin αvβ3 is overexpressed in cancer and implicated in cancer progression and tumor angiogenesis [6]. Integrin αvβ3 plays a critical role in regulating IGF1 signaling. Antagonists to αvβ3 block IGF1 signaling [7,8] and it has thus been well established that the αvβ3 integrin is critical for IGF1 signaling. The role of αvβ3 in IGF1 signaling, however, has not been fully established. We discovered that IGF1 directly binds to αvβ3 and induces the αvβ3-IGF1-IGF1R ternary complex [9]. The IGF1 mutant (the R36E/R37E mutant) in the predicted integrin-binding site of IGF1 in the C-domain is defective in integrin binding and in inducing IGF signaling, although the mutant still binds to IGF1R [9]. These findings suggest that the binding of integrins to IGF1 is critical for IGF1 signaling. Notably, the R36E/R37E IGF1 mutant suppresses anchorage-independent growth of cancer cells (dominant-negative effects) in vitro and tumorigenesis in vivo, while WT IGF1 markedly enhances these processes [10], although the role of αvβ3 is not clear [11].

Gα13 is a guanine nucleotide-binding protein of the G12 class of Gα proteins. Gα13 activates RhoA by binding and activating RhoGEFs [12] and regulates cytoplasmic as well as nuclear signaling events such as activation of the Jun N-terminal kinase signaling module, Na^+^/H^+^ exchangers, and focal adhesion assemblies, as well as transcriptional activation of specific primary response genes. Like Gα12, Gα13 is up-regulated in several human cancers, and Gα12/Gα13 signaling plays an important role in cancer cell invasion and metastasis. Gα13 mediates outside-in signaling by binding to the cytoplasmic domain of integrin β3 through the EEE motif (731-733 of β3) [13,14,15]. The Gα13-β3 interaction is promoted by ligand binding to integrin αIIbβ3, which leads to RhoA activation [13].

In the present study, we first show that a mutation in the β3 cytoplasmic domain (EEE to AAA) of αvβ3 suppressed IGF1 signaling, suggesting that Gα13 binding to the β3 tail is involved in IGF1 signaling. We hypothesized that Gα13 binds to and activates signaling molecules other than RhoA, since RhoA activation may not induce IGF1R activation. Docking simulations predict that Gα13 binds to the IGF1R kinase domain. Neither the IGF1R binding site in Gα13 nor the Gα13 binding site in the IGF1R kinase has not been shown to interact with other molecules. Notably, mutating the predicted IGF1R kinase binding site of Gα13 (residues 260-279) to Ala suppressed IGF1 signaling. Taken together, we propose that IGF1 binding to αvβ3 induces Gα13 binding to the β3 tail and triggers Gα13 binding to the IGF1R kinase (outside-in signaling) through the novel binding sites.

## 2. Results

### 2.1. Gα13 Binding to the β3 Tail Is Involved in Outside-In Signaling and IGF1 Signaling

**Figure 1:**
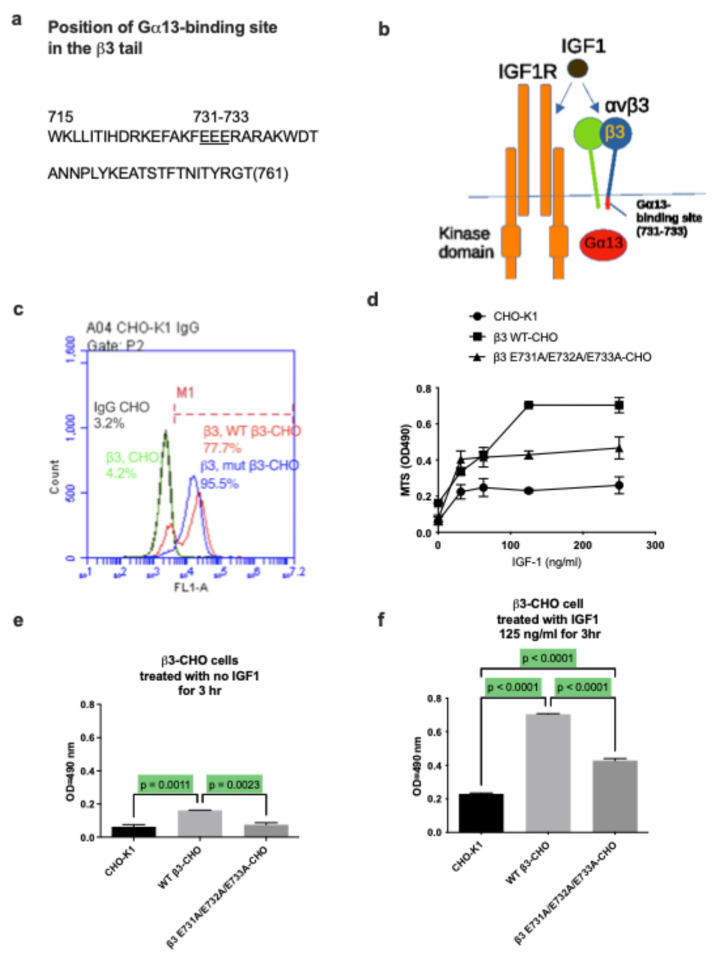
The β3 mutant (EEE 731-733 to AAA) defective in Gα13 binding to the β3 tail suppresses cell survival induced by IGF1. (**a**) Position of the Gα13 binding site in the β3 tail [15]. (**b**) We hypothesize that IGF1 binding to integrin αvβ3 induces Gα13 binding to the β3 tail through the EEE motif, and this process is critical for IGF1 signaling. To prove this hypothesis, we tested if the β3 mutant defective in Gα13 binding affects IGF1 signaling. (**c**) WT β3 or the β3 EEE-to-AAA mutant [15] was stably expressed in CHO cells (low in β3), and cells were sorted for human β3 expression with anti-human β3. WT β3-CHO (77.7%) (red); mutant human β3-CHO (95.5%) (blue); CHO cells with anti-human β3 (4.2%) (green); parent CHO cells with control mouse IgG (3.2%) (black). The expression of WT and mutant human β3 was comparable. (**d**–**f**) Cells were serum-starved and incubated with or without IGF1 in polyHEMA-coated wells for 24 h. Dose response of IGF1-induced cell survival of CHO cells expressing WT and mutant β3 (**d**). Cell viability was measured using MTS assays. Cell survival without IGF1 (**e**) or with IGF1 (125 ng/mL) (**f**).

IGF1 directly binds to integrin αvβ3 and induces the αvβ3-IGF1-IGF1R ternary complex on the surface [9]. WT IGF1 induces robust survival signals, but the IGF1 R36E/R37E mutant defective in integrin binding did not [9]. CHO cells (β3-deficient, IGF1R+) that over express human β3 (β3-CHO cells) respond to IGF1 in an αvβ3-dependent manner, but parent CHO cells did not [11], suggesting that IGF1 signaling is dependent on integrin αvβ3. These findings suggest that IGF1 signaling is dependent on IGF1 binding to αvβ3 in CHO cells. Gα13 binding to the β3 tail is predicted to mediate outside-in signaling from integrin αvβ3 upon ECM binding, and the β3 mutation (EEE to AAA, residues 721-723 of β3) (Figure 1a) inhibits Gα13 binding to the β3 tail [15]. We hypothesized that IGF1 binding to αvβ3 induces Gα13 binding to the β3 tail, and Gα13 mediates downstream IGF1 signaling (Figure 1b). To address this hypothesis, WT or β3 (EEE/AAA) mutants were stably expressed in CHO cells, and β3-positive cells were enriched by sorting. The levels of β3 expression were comparable in the two populations expressing WT or mutant β3 (Figure 1c). Cells were serum-starved, plated in anchorage-independent conditions (on polyHEMA-coated wells) and treated with IGF1 for 24 h (Figure 1d). Negative controls, that is, cells that are not transfected with human β3 or not treated with IGF1, showed much less response (Figure 1e). CHO cells transfected with WT β3 and treated with IGF1 showed a strong response, and cells expressing the β3 EEE-to-AAA mutant showed much less response to IGF1 (Figure 1f). These findings suggest that Gα13 binding to the β3 tail is required for IGF1 signaling.

### 2.2. Docking Simulation Predicts That Gα13 Binds to the IGF1R Kinase

**Figure 2:**
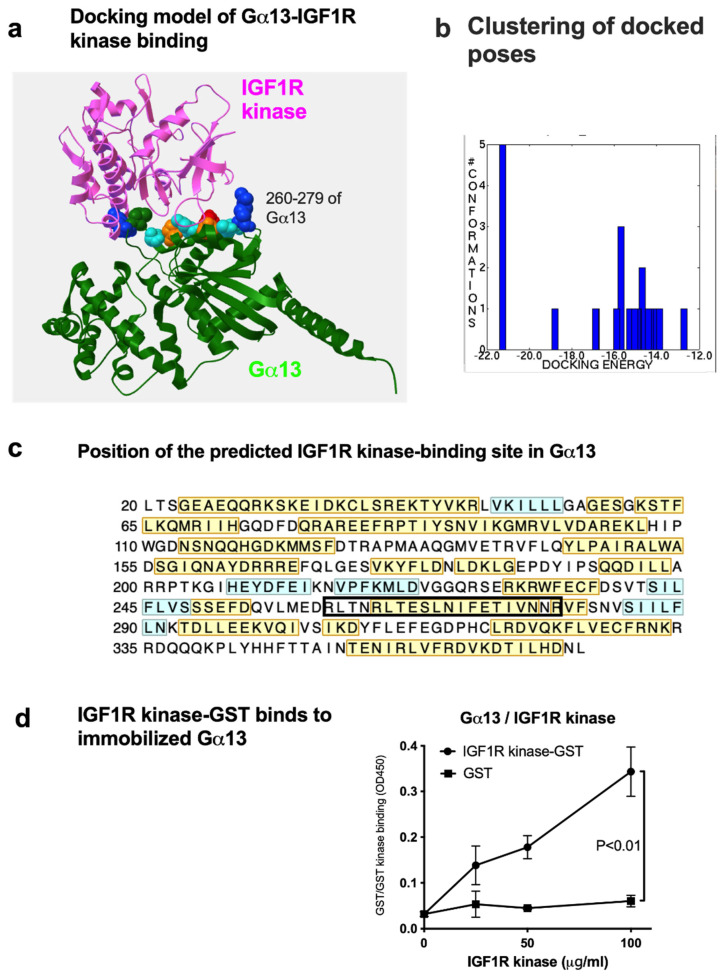
Interaction between Gα13 and the IGF1R kinase. We studied if Gα13 binds to the IGF1R kinase by a docking simulation. The simulation predicts that (1) the IGF1R kinase binds to Gα13 well and that (2) the binding interface is not in the catalytic sites of either molecule. (**a**) A model of the Gα13/IGF1R kinase complex. Residues 260-279 of Gα13 are involved in binding to the IGF1R kinase. (**b**) Clustering analysis of docked poses between Gα13 and the IGF1R kinase (PDB code: 3QQU). The model in cluster 1 was further analyzed. (**c**) Position of the predicted IGF1R binding site in Gα13. Amino acids in yellow are in helices. Amino acid residues in blue are in loops. Amino acid residues in black rectangles (residues 260-279) are involved in IGF1R binding. (**d**) Gα13 and IGF1R interact in ELISA-type binding assays. Mouse Gα13 (1 μg/mL) was immobilized to wells in a 96-well microtiter plate and incubated with the IGF1R kinase (GST fusion). The data suggests that the IGF1R kinase interacts with Gα13 in a dose-dependent manner. The control GST did not bind to Gα13. Data is shown as means +/− SD of triplicate experiments.

**Figure 3:**
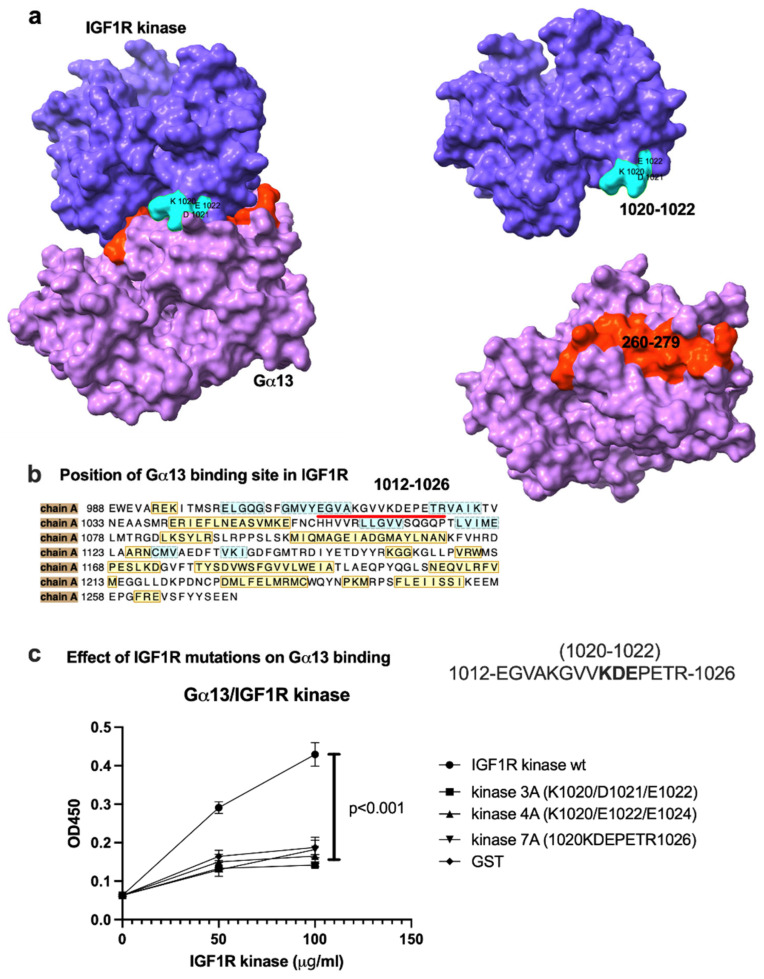
Mutations in the predicted Gα13 binding site of the IGF1R kinase (residues 1012-1026) inhibit IGF1R kinase binding to Gα13. (**a**) Docked model of Gα13-IGF1R interactions. (**b**) Positions of the predicted amino acids of the IGF1 kinase involved in Gα13 binding. Amino acids in yellow are in helices. Amino acid residues in blue are in loops. Amino acid residues in black rectangles (residues 1012-1026) are involved in Gα13 binding. Amino acid residues in the predicted IGF1R kinase binding interface of Gα13 were selected for mutagenesis. We changed three to seven residues in the Gα13 binding interface to Ala. (**c**) Effect of IGF1R kinase mutations on Gα13 binding. The IGF1R kinase mutants were tested for Gα13 binding using ELISA-type binding assays as described in the Materials and Methods Section. Wells in a 96-wells microtiter plate were coated with Gα13 (1 μg/mL in PBS) and incubated with the WT IGF1R kinase or mutants, and we quantified the bound IGF1R kinase using HRP-conjugated anti-GST. Data is shown as means +/− SD of triplicate experiments.

It is unclear if Gα13 activates signaling molecules other than RhoA. Since Gα13 is known to bind to the Src tyrosine kinase [16], Btk, or Pyk2 cytosolic tyrosine kinases and activates them [17,18], we hypothesized that Gα13 binds to and activates the tyrosine kinase domain of IGF1R. A docking simulation of the interaction between the IGF1R kinase and Gα13 predicts that Gα13 binds to the IGF1R kinase (3QQU.pdb) (Figure 2a). Clustering of docked poses suggests that cluster 1, with the lowest docking energy (docking energy −21.3 kcal/mol), is likely the pose in which IGF1R binds to Gα13 (Figure 2b). Amino acid residues in Gα13 and the IGF1R kinase are predicted to be involved in interactions and are shown in Table 1 and Figure 2c. The Src tyrosine kinase showed much lower affinity to Gα13 (−16.9 kcal/mol) than the IGF1R kinase. We studied if Gα13 directly binds to the IGF1R kinase via ELISA-type binding assays. The IGF1R kinase fused to GST was incubated with wells coated with the Gα13 protein, and the bound kinase domain was quantified using HRP-conjugated anti-GST. The IGF1R kinase bound to Gα13 in a dose-dependent manner (Figure 2d). This suggests that Gα13 interacts with the IGF1R kinase. The positions of Gα13 and the IGF1R kinase binding interface are shown in Figure 3a. We introduced point mutations of several residues in the predicted Gα13 binding interface (residues 1020-1026) to Ala in the IGF1R kinase (Figure 3b). The mutations effectively suppressed IGF1R kinase binding to Gα13 (Figure 3c). The minimum mutation (residues K1020A/D1021A/E1022A) (kinase 3A) was sufficient for blocking IGF1R kinase binding to Gα13 (Figure 3c). These findings are consistent with the docking model.

### 2.3. The Gα13 Variant with the 260-279A Mutation in the IGF1R Kinase Binding Site Is Defective in IGF1-Induced Cell Survival

**Figure 4:**
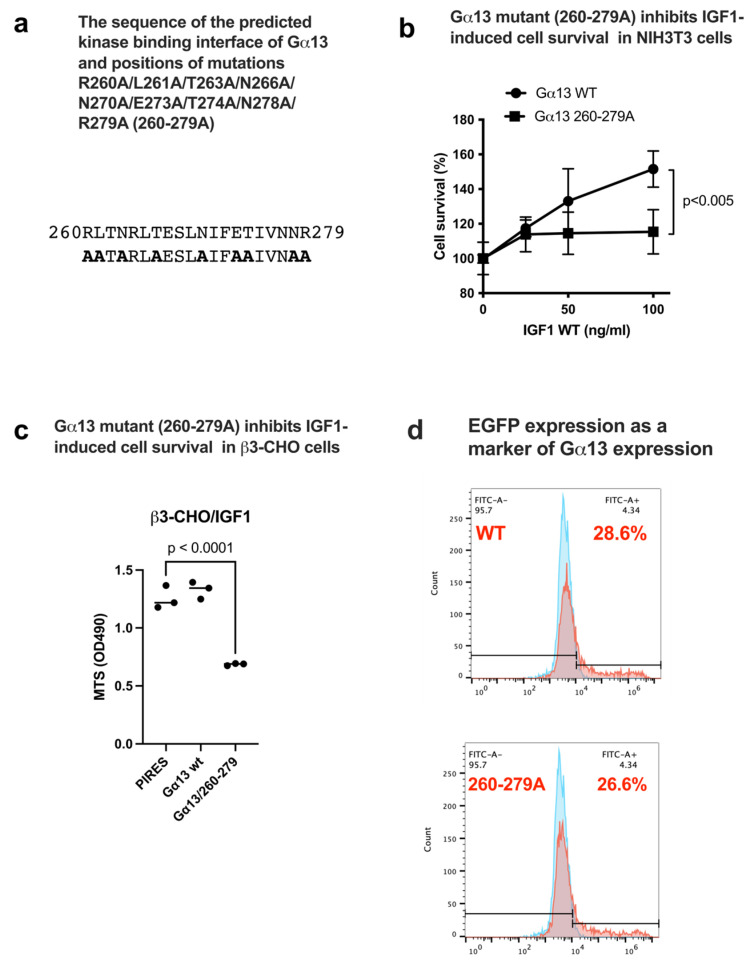
Expression of the Gα13 260-279A mutant suppresses IGF1-induced cell survival in NIH 3T3 cells. (**a**) The predicted IGF1R kinase binding interface of Gα13 (residues 260-279) and positions of mutations. Only the amino acid residues exposed to the surface were selected for mutagenesis. (**b**) Transient expression of the Gα13 260-279A mutant suppressed cell survival induced by IGF1 in NIH3T3 cells. NIH3T3 cells were transfected with WT Gα13 and the 260-279A mutant using the pcDNA3.1 vector. Two days after transfection in a regular medium, cells were divided into 3 wells (coated with polyHEMA) in serum-free DMEM and treated with IGF1 (up to 100 ng/mL) under anchorage-independent conditions (polyHEMA). Cell survival was measured using MTS assays. (**c**) β3-CHO cells were transiently transfected with WT Gα13 and the 260-279 mutant using the bicistronic PIRES-EGFP-puro vector. Two days after transfection with Gα13, cells were divided into 3 wells (coated with polyHEMA) in serum-free DMEM and treated with 100 ng/mL IGF1. Cell survival was measured using MTS assays. (**d**) EGFP expression from the PIRES-EGFP-puro vector was measured in a flow cytometer and used as a marker of Gα13 expression (26–28%) (red). Control non-transfected β3-CHO cells (blue, 4.3%). Data are shown as means +/− SD of triplicate experiments.

Mutations were introduced in the predicted IGF1R binding interface in Gα13 (residues 260-279 of Gα13). Only surface-exposed amino acid residues were mutated to Ala (R260/L261/N263/T266/N270/E273/T274/N278/R279 to A, designated Gα13 260-279A) (Figure 4a). We studied if non-transformed NIH3T3 cells, which respond well to IGF1, are affected by the Gα13 260-279A mutation. We transiently expressed WT Gα13 or the 260-279A mutant in pcDNA3.1 to cells and cultured with IGF1 (100 ng/mL) under serum-free and anchorage-independent conditions. The Gα13 260-279A mutant suppressed cell survival induced by IGF1 (Figure 4b), suggesting that the Gα13 260-279A mutant is defective in enhancing IGF1-induced cell survival in non-transformed cells. These findings are consistent with the docking model, and Gα13 binding to the IGF1R kinase is involved in IGF1 signaling.

The WT and mutant Gα13 variants were transiently expressed in β3-CHO cells. WT Gα13 did not further enhance cell survival induced by IGF1 probably due to endogenous Gα13. Gα13 260-279A suppressed cell survival via IGF1 (Figure 4c). These findings suggest that IGF1 binding to αvβ3 effectively activates Gα13 in β3-CHO cells, and Gα13 260-279A suppresses Gα13 binding to the IGF1R kinase. In this experiment, we used the IRES2-EGFP bicistronic vector [19,20], in which both EGFP and Gα13 (WT and mutants) are encoded in a single mRNA with two separate translation starting sites. EGFP expression, which serves as a marker of Gα13 expression, was comparable among the transfected cells used (Figure 4d).

### 2.4. The Oncogenic Gain-of-Function Q226L Mutant Enhances Cell Survival in β3-CHO Cells Without IGF1 and Its Effect Is Suppressed by the 260-279 Mutation

**Figure 5:**
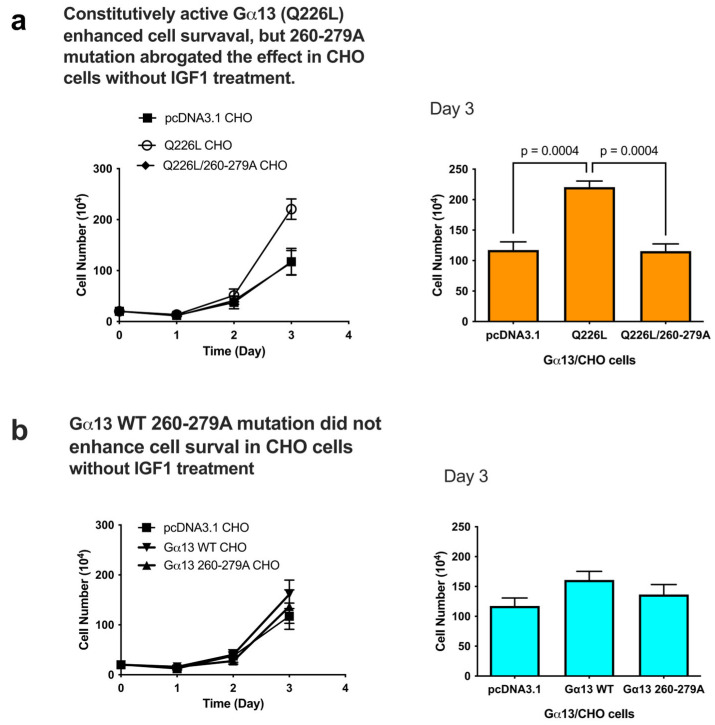
The Gα13 Q226L mutant enhanced survival and the 260-279 mutant suppressed Q226L-induced cell proliferation in CHO cells. CHO cells were transiently transfected with Gα13 Q226L or Q226L/260-279A double mutants using the pcDNA3.1 vector, and cell proliferation was measured by counting cell numbers. The data suggest that the 260-279A mutation abrogated the effect of Gα13 Q226L. Data are shown as means +/− SD of triplicate experiments.

The constitutively active Gα13 mutant Q226L is a potent oncogene [21], but its mechanism of action is unclear. The Gα13 Q226L mutant enhanced cell survival in CHO cells (β3 low), but Q226L/260-279A double mutants did not (Figure 5). IGF1 was not included. The data suggest that the 260-279A mutant abrogated the effect of Q226L. We suspect that the Q226L mutant might enhance binding to the IGF1R kinase, and the mutation in the kinase binding site (260-279A mutation) inhibits Gα13 binding to the IGF1R kinase and thereby suppresses enhanced cell survival by the Q226L mutation.

**Figure 6:**
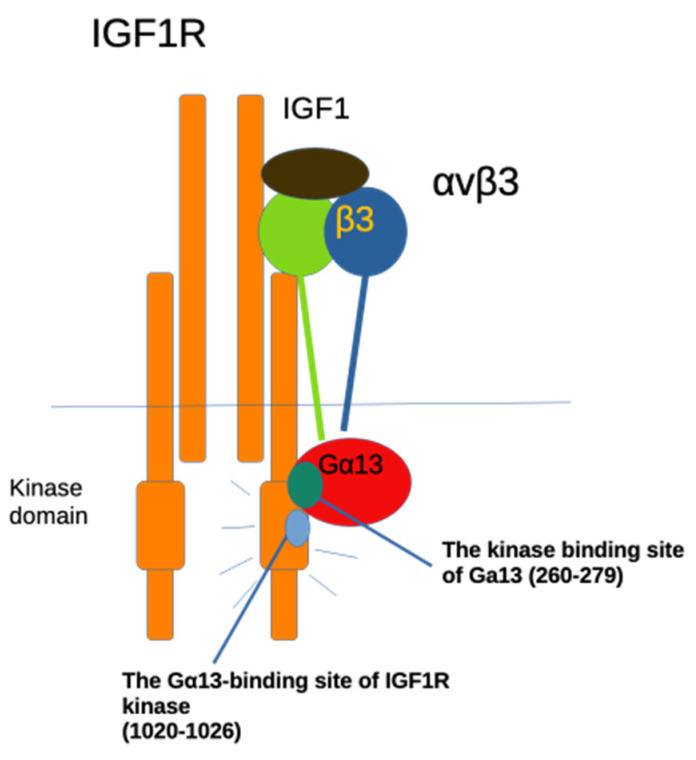
A potential role of Gα13 in IGF1 signaling. Previous studies showed that Gα13 binds to the β3 cytoplasmic domain upon ECM ligand binding to αvβ3 through the EEE motif and subsequently induces RhoA activation. Also, we previously showed that IGF1 binds to αvβ3 and induces the αvβ3-IGF1-IGF1R ternary complex. The present study showed that mutating the EEE motif of the β3 tail suppressed IGF1-induced cell survival, suggesting that Gα13 is involved in IGF1 signaling. We showed that mutating the predicted IGF1R binding site of Gα13 (residues 260-279) suppressed Gα13 binding to IGF1R kinase. This mutation suppressed IGF1 signaling (IGF1-induced enhanced cell survival), suggesting that Gα13 binding to the IGF1R kinase domain is involved in IGF1 signaling. Also, the Gα13 260-279 mutation suppressed constitutive activation of Gα13 (Q226L), suggesting that this Q226L mutation enhances Gα13 binding to the IGF1R kinase and that the mutation in the kinase binding site of Gα13 abrogates the effect of the Q226L mutation. A mutation of the predicted Gα13 binding site in the IGF1R kinase (residues 1020-1022) suppressed Gα13 binding to Gα13, which is consistent with the docking model. When ECM ligands bind to αvβ3, Gα13 binds to the β3 tail, but it is unclear if IGF1R kinases are activated. This is because ECM ligands do not induce ternary complex formation with integrin and IGF1R, and the IGF1R kinase domain is physically not close to αvβ3. The horizontal line represents cell membrane.

Taken together, we propose that IGF1 binding to integrin αvβ3 effectively triggers Gα13 binding to the IGF1R kinase. The Gα13 260-279 mutation inhibits the binding of Gα13 or Gα13 Q226L to the IGF1R kinase and subsequent kinase activation (Figure 6).

## 3. Discussion

In the present study, we showed that Gα13 binding to the β3 tail is critically involved in IGF1 signaling. Gα13 binding to the β3 tail is known to activates RhoA, but it is unknown if Gα13 binding to the β3 tail induces any other signaling pathways. We propose that Gα13 binds to the IGF1R kinase domain. Notably, the IGF1R kinase binding site in Gα13 (residues 260-279) is not the Gβ or Gγ binding site. The Gα13 260-279 A mutation inhibited IGF1-induced cell survival, suggesting that Gα13 binding to the IGF1R kinase may be critical for IGF1 signaling. We also showed that mutations in the Gα13 binding site in the IGF1R kinase suppressed Gα13 binding, suggesting that the docking model is correct.

The predicted binding site of Gα13 in the IGF1R or IGF1R kinase-binding site in Gα13 has not been shown to bind to any signaling molecules or to have catalytic activity. The Gα13 binding site in the IGF1R kinase is not involved in catalytic action, Tyr phosphorylation, or Gβ/Gγ binding. It is likely that Gα13 activates IGF1R as a downstream effector and induces cell proliferation. Also, the Gα13 260-279A mutation abrogated constitutive activation of the Gα13 Q226L mutant. We suspect that the Q226L mutation may enhance the binding of Gα13 to the IGF1R kinase, and this may be related to its oncogenic action. IGF1R is frequently overexpressed in many cancers and is a major target for anti-cancer therapies. Mutating the predicted Gα13 binding site in IGF1R (residues 1020–1022 to A) inhibited Gα13 binding to the IGF1R kinase, consistent with the proposed role of the region in Gα13 binding.

It has been proposed that IGF1 binding to IGF1R (ectodomain) induces conformational changes in IGF1R and thereby activates the kinase domain inside the cells [22]. IGF1R is already a disulfide-linked α2β2 tetramer, and therefore classical ligand-induced dimerization of receptors may not fit in IGF1R kinase activation. The IGF1 mutant (R36E/R37E) is defective in binding to integrin αvβ3 and signaling function (IGF1R activation), although the mutant still binds to IGF1R [9], suggesting that IGF1 binding to αvβ3 is required for subsequent Gα13 binding to the IGF1R kinase. These findings suggest that IGF1 binding to IGF1R may not be sufficient for IGF1R activation, and IGF1 binding to αvβ3 and subsequent Gα13 binding to IGF1R kinase are critical for IGF1R activation. The present study suggests that IGF1 binding to αvβ3 induces Gα13 binding to the β3 tail and subsequent Gα13 binding to the IGF1R kinase, resulting in IGF1R activation (Figure 6).

Gα13 may be fully activated in β3-CHO cells upon IGF1 stimulation (as in the Gα13 Q226L cells) and has a high affinity for the IGF1R kinase. Notably, Gα13 260-279A effectively suppressed cell survival induced by Q226L, suggesting that the Q226L mutant may have a high affinity for the IGF1R kinase (like activated Gα13); in contrast, the 260-279A mutant is defective or has a low affinity for the IGF1R kinase. The present study explains why high-level αvβ3 enhances cell proliferation in cancer. It is likely that high-level αvβ3 facilitates Gα13 activation. Blocking Gα13 binding to the IGF1R kinase (e.g., using Gα13 or IGF1R kinase mutants) would be potentially useful for suppressing cancer proliferation. There is, however, a limitation of this study. The interaction between the IGF1R kinase and Gα13 is primarily supported by in silico modeling and in vitro biochemical assays, and direct validation of the protein–protein interaction in cells is still required to confirm the proposed model in future studies.

## 4. Materials and Methods

### 4.1. Gα13 Constructs

Wild-type Gα13 (EE-tagged) and Q226L cDNA in pcDNA3.1 were obtained from the UMR cDNA Resource Center, University of Missouri-Rolla (Rolla, MO, USA).

### 4.2. Synthesis of the RTK Kinase Domains

A cDNA fragment encoding the kinase domain (residues 999-1274 for IGF1R) was synthesized and subcloned into the Bam HI/Eco RI site of the pGEX2T vector. Protein was synthesized in *E. coli* BL21 and purified by glutathione–Sepharose affinity chromatography.

### 4.3. ELISA-Type Binding Assay

Wells in a 96-well microtiter plate were incubated with recombinant mouse Gα13 (1 μg/mL) in PBS for 1h at room temperature, and the remaining binding sites were blocked by incubating with 0.1% heat-treated BSA (80 °C for 10 min). Wells were incubated with the IGF1R kinase domain (fused to GST) in PBS for 1 h at room temperature. Control GST was used as a negative control. After washing the wells, the bound IGF1R kinase was quantified using HRP-conjugated anti-GST and a peroxidase substrate.

### 4.4. Effect of the β3 Mutant (EEE 731-733 to AAA) Defective in Gα13 Binding on Cell Survival Induced by IGF1

WT β3 or β3 EEE-to-AAA mutants [15] were stably expressed in CHO cells, and cells were sorted for β3 expression. The expression of WT and mutant β3 were comparable. Cells were serum-starved and incubated with or without IGF1 (125 ng/mL) in polyHEMA-coated wells for 24 h. Cell viability was measured using MTS assays.

### 4.5. Effect of the Gα13 260-279A Mutant on Survival of β3-CHO Cells Induced by IGF1

β3-CHO cells were transfected with WT Gα13 and the 260-279 mutant using the bicistronic PIRES-EGFP-puro vector. Two days after transfection of Gα13 in the bicistronic PIRES vector in a regular medium, cells were divided into 3 wells (coated with polyHEMA) in serum-free DMEM and treated with 100 μg/mL IGF1. Cell survival was measured using MTS assays. EGFP expression from the PIRES-EGFP-puro vector was used as a marker of Gα13 expression.

Site-directed mutagenesis was performed using the QuickChange method [23].

### 4.6. Docking Simulation

The Gα13 structure was obtained from the Swiss model (Q14344_47-368:3ab3.2.A), since the original structures from the protein data bank (PDB) have missing parts. Since the whole Gα13 model (residues 47-379) is too big for docking analysis, the structure was divided into two parts, and the C-terminal part (residues 203-369) was predicted to bind to the IGF1R kinase. A docking simulation between the C-terminal region of Gα13 and the IGF1R kinase (3QQU.pdb) was performed using Autodock 3.05 [23]. The ligand was then compiled to a maximum size of 3072 atoms. Atomic solvation parameters and fractional volumes were assigned to the protein atoms using the AddSol tool, and grid maps were calculated using the AutoGrid tool in AutoDock 3.05. A grid map with 127 × 127 × 127 points and a grid point spacing of 0.603 Å were included the headpiece of αvβ3. Kollman ‘united-atom’ charges were used. AutoDock 3.05 uses a Lamarckian genetic algorithm (LGA) that couples a typical Darwinian genetic algorithm for global searching with the Solis and Wets algorithm for local searching. The LGA parameters were defined as follows: the initial population of random individuals had a size of 50 individuals; each docking step was terminated with a maximum number of 1 × 10^6^ energy evaluations or a maximum number of 27,000 generations, whichever came first; and mutation and crossover rates were set at 0.02 and 0.80, respectively. An elitism value of 1 was applied, which ensured that the top-ranked individual in the population always survived to the next generation. A maximum of 300 iterations per local search was used. The probability of performing a local search on an individual was 0.06, whereas the maximum number of consecutive successes or failures before doubling or halving the search step size was 4. Clustering analysis was performed using Autodocktools v1.57. We selected amino acid residues in the predicted binding interface (shown in Table 1). We then changed them to Ala.

### 4.7. Statistical Analysis

We used Prism 10 (Graphpad Software, Boston, MA, USA) to test treatment differences, and ANOVA and Tukey multiple comparison tests were used to control the global type I error.

## 5. Conclusions

IGF1 directly binds to integrin αvβ3 and induces the αvβ3-IGF1-IGF1R ternary complex, but the role of αvβ3 has not been fully established. Gα13 is shown to be involved in integrin-induced outside-in signaling by binding to the β3 tail. Gα13 binding to β3 results in RhoA activation. Our studies show that the β3 tail with mutations in the Gα13 binding site (EEE to AAA) suppressed IGF1 signaling, suggesting that Gα13 binding to β3 is required for IGF1 signaling. Previous studies show that Gα13 binding to the β3 tail results in RhoA activation, but they did not show whether IGF1R is activated by RhoA activation. Docking simulation between Gα13 and the kinase domain of IGF1R predicted that Gα13 binds to the IGF1R kinase domain. Gα13 bound to the IGF1R kinase domain in ELISA in a dose-dependent manner. Gα13 with mutations in the predicted kinase binding site suppressed the enhanced IGF1-induced cell survival of β3-CHO cells, indicating that Gα13-IGF1R kinase interaction is critical for IGF1 signaling. Also, a mutation of the predicted Gα13 binding site in the IGF1R kinase domain suppressed Gα13 binding, consistent with the docking model. We hypothesize that integrin–IGF1–IGF1R ternary complex formation induces IGF1R activation through Gα13. We propose that IGF1 binding to αvβ3 induces the αvβ3-IGF1-IGF1R complex and subsequently Gα13 binding to the β3 tail. This will lead to Gα13 binding to the kinase domain of IGF1R and IGF1R activation. Our studies provided a new potential outside-in signal transduction pathway from the integrin–αvβ3–IGF1–IGF1R ternary complex through Gα13 binding to the β3 tail. Also, the Gα13 (260-279A) mutation defective in IGF1R kinase binding abrogated cell proliferation by the oncogenic Gα13 Q226L mutation, suggesting that Q226L’s action includes enhanced Gα13 binding to the IGF1R kinase. There is, however, a limitation of this study. The interaction between the IGF1R kinase and Gα13 is primarily supported by in silico modeling and in vitro biochemical assays, and direct validation of the protein–protein interaction in cells is still required to confirm the proposed model in future studies.

## Figures and Tables

**Table 1:** Amino acid residues involved in Gα13-IGF1R kinase interactions, as predicted by the docking simulation.

Gα13 (−21.25 kcal/mol)	IGF1R Kinase (3QQU.pdb)
Arg227, Ser228, Glu229, Arg230, Lys231, Trp233, Phe234, Val255, Glu258, Asp259, **Arg260, Leu261, Thr262, Asn263, Thr266, Glu267, Leu269, Asn270, Glu273, Thr274, Ile275, Asn277, Asn278, Arg279,** Val280, Lys306, Asp307, Tyr308, Phe309, Leu310, Glu311, Lys333, Arg335, Asp336	Thr997, Met998, Ser999, Arg1000, Glu1012, Gly1013, Val1014, Lys1016, Lys1020, Asp1021, Glu1022, Pro1023, Glu1024, Thr1025, Arg1026, His1057, His1058, Thr1080, Arg1081, Tyr1087, Leu1091, Pro1104, Ser1105, Lys1108, Gln1111, Glu1115, Glu1142, Asp1143, Phe1144, Thr1145, Glu1268, Pro1269, Gly1270, Phe1271, Glu1273, Val1274

Amino acid residues within 0.6 nm of Gα13 and the IGF1R kinase were selected using Pdb Viewer (version 4.1). Amino acid residues of Gα13 selected for mutation are shown in bold. Amino acid residues of the IGF1R kinase selected for mutation are underlined.

## Data Availability

The original contributions presented in this study are included in the article. Further inquiries can be directed to the corresponding author.
